# Transcriptional responses in jejunum of two layer chicken strains following variations in dietary calcium and phosphorus levels

**DOI:** 10.1186/s12864-021-07814-9

**Published:** 2021-06-29

**Authors:** Henry Reyer, Michael Oster, Siriluck Ponsuksili, Nares Trakooljul, Adewunmi O. Omotoso, Muhammad A. Iqbal, Eduard Muráni, Vera Sommerfeld, Markus Rodehutscord, Klaus Wimmers

**Affiliations:** 1 Leibniz Institute for Farm Animal Biology (FBN), Institute for Genome Biology, Wilhelm- Stahl-Allee 2, 18196 Dummerstorf, Germany; 2 Institute of Animal Science, University of Hohenheim, Emil-Wolff-Str. 10, 70599 Stuttgart, Germany; 3 Faculty of Agricultural and Environmental Sciences, University Rostock, Justus-von-Liebig- Weg 7, 18059 Rostock, Germany

**Keywords:** laying hen line, mineral requirements, mineral homeostasis

## Abstract

**Background:**

Calcium (Ca) and phosphorus (P) are essential nutrients that are linked to a large array of biological processes. Disturbances in Ca and P homeostasis in chickens are associated with a decline in growth and egg laying performance and environmental burden due to excessive P excretion rates. Improved utilization of minerals in particular of P sources contributes to healthy growth while preserving the finite resource of mineral P and mitigating environmental pollution. In the current study, high performance Lohmann Selected Leghorn (LSL) and Lohmann Brown (LB) hens at peak laying performance were examined to approximate the consequences of variable dietary Ca and P supply. The experimental design comprised four dietary groups with standard or reduced levels of either Ca or P or both (*n* = 10 birds per treatment group and strain) in order to stimulate intrinsic mechanisms to maintain homeostasis. Jejunal transcriptome profiles and the systemic endocrine regulation of mineral homeostasis were assessed (*n* = 80).

**Results:**

Endogenous mechanisms to maintain mineral homeostasis in response to variations in the supply of Ca and P were effective in both laying hen strains. However, the LSL and LB appeared to adopt different molecular pathways, as shown by circulating vitamin D levels and strain-specific transcriptome patterns. Responses in LSL indicated altered proliferation rates of intestinal cells as well as adaptive responses at the level of paracellular transport and immunocompetence. Endogenous mechanisms in LB appeared to involve a restructuring of the epithelium, which may allow adaptation of absorption capacity via improved micro-anatomical characteristics.

**Conclusions:**

The results suggest that LSL and LB hens may exhibit different Ca, P, and vitamin D requirements, which have so far been neglected in the supply recommendations. There is a demand for trial data showing the mechanisms of endogenous factors of Ca and P homeostasis, such as vitamin D, at local and systemic levels in laying hens.

**Supplementary Information:**

The online version contains supplementary material available at 10.1186/s12864-021-07814-9.

## Background

Sufficient dietary supply of calcium (Ca) and phosphorus (P) is essential for all vertebrates to ensure various biological processes including bone formation, blood clotting, cell proliferation and energy metabolism. In avian species, the egg laying phase in general and high laying rates in particular generate extra demands on mineral homeostasis and nutrient flows. The continous process of eggshell formation and yolk production during the laying period requires high amounts of dietary Ca [1]. In fact, Ca accounts for 40 % of the eggshell weight in the form of CaCO_3_. The sources of Ca and P for laying hens are derived from mineral supplements and plant-derived compounds. However, depending on the feedstuff components, up to 80 % of P occurs in the form of inositol phosphates with a considerable variation in their abundance [2, 3], which are available for intestinal absorption only following enzymatic cleavage [4]. Therefore, phytases of microbial origin are added to the feed to increase the intestinal availability [5]. In addition, diets of highly productive laying hen strains are supplemented with high-quality inorganic phosphates to meet required levels of dietary available P or nonphytate P. The inefficient use of P makes monogastric animal species significant P excretors and thus a major source of P input into the environment [6]. To reduce the environmental impact of animal production and to preserve the valuable natural resources of P, measures on digestibility and nutrient utilization are needed to increase the use of plant P taking into account management strategies and animal-based approaches.

In vertebrates, mechanisms of P homeostasis are largely conserved and closely linked to Ca metabolism. In particular the dietary Ca/P ratio has to meet physiological ranges and has a strong impact on health and performance data [7]. Due to the stoichiometric equilibrium of Ca and P and the tight regulation of the Ca/P ratio in serum and body fluids, measures to maintain mineral homeostasis during the laying period will affect both minerals. This includes absorption, storage and excretion processes at the level of gastrointestinal tract, bone, and kidney, which are strictly controlled by a number of known and as yet unknown regulators, transporters and endocrine and paracrine signals. Key regulators are the parathyroid hormone (PTH), the active form of vitamin D3 (calcitriol), calcitonin and fibroblast growth factor 23 (FGF23). PTH is synthesized by the parathyroid glands and its secretion depends largely on the Ca concentration in serum, which is sensed by the Ca-sensing receptor (CASR) [8]. Downstream functions of PTH comprise the short-term and sustained activation of molecular pathways that are involved in maintaining serum Ca levels mainly via improved bone resorption and renal Ca reabsorption, while enhancing renal P excretion [9]. Moreover, PTH receptors have been detected in the duodenum of chickens where they mediate a direct effect on intestinal Ca transport and influence P absorption processes [10]. In general, intestinal Ca and P absorption is achieved via para- and trans-cellular transport processes, which are responsive to dietary mineral supply [11]. In particular, the jejunum and duodenum are considered to be the primary sites of P absorption in the gastrointestinal tract [12]. Vitamin D3 controls Ca and P homeostasis through direct actions on the intestine, kidney, and bones and through feedback inhibition of PTH production in the parathyroid. These actions are mainly mediated by binding of the activated vitamin D receptor to vitamin D response elements (VDRE) in the promoter regions of various target genes [13]. Regarding laying hens, it has been shown that Ca and P utilization are strongly dependent on vitamin D [14]. FGF23, which is derived from osteoblasts and osteocytes, affects serum concentrations of P and PTH, as well as renal P transporter expression and the formation of active vitamin D in the kidney [15].

The laying performance of commercial laying hens is exceptionally high and requires high dietary standards, especially with regard to mineral supply. Two important representative layer strains are Lohmann Selected Leghorn (LSL) and Lohmann Brown (LB) with a similar laying performance over the production period [16]. Nevertheless, there are distinct differences between the two strains in terms of body weight and immunity as well as in bone metabolism and phytate degradation [17–19]. The current study is based on a previous experimental trial using high performing laying hens of these distinct genetic origins (LSL and LB) to investigate the dietary impact of variable Ca and P supply [19]. This study extends the previous investigations by assessing an endocrine and metabolic pattern in plasma as well as molecular transcriptional responses at the level of the small intestine obtained by RNA sequencing. Specifically, the proximal part of the jejunum, as the main site of mineral absorption, is the focus of the study. This approach aims to elucidate the jejunal contribution to the complex regulation of mineral homeostasis in individuals at peak performance. It is hypothesized that diets low in P and/or Ca trigger phenotypic and molecular adaptations in laying hens to orchestrate e.g. mineral absorption and storage in order to maintain mineral homeostasis. Strain-specific transcriptional responses can identify genotype-environment interactions to be incorporated into strategies for targeted resource management.

## Results

**Fig. 1:**
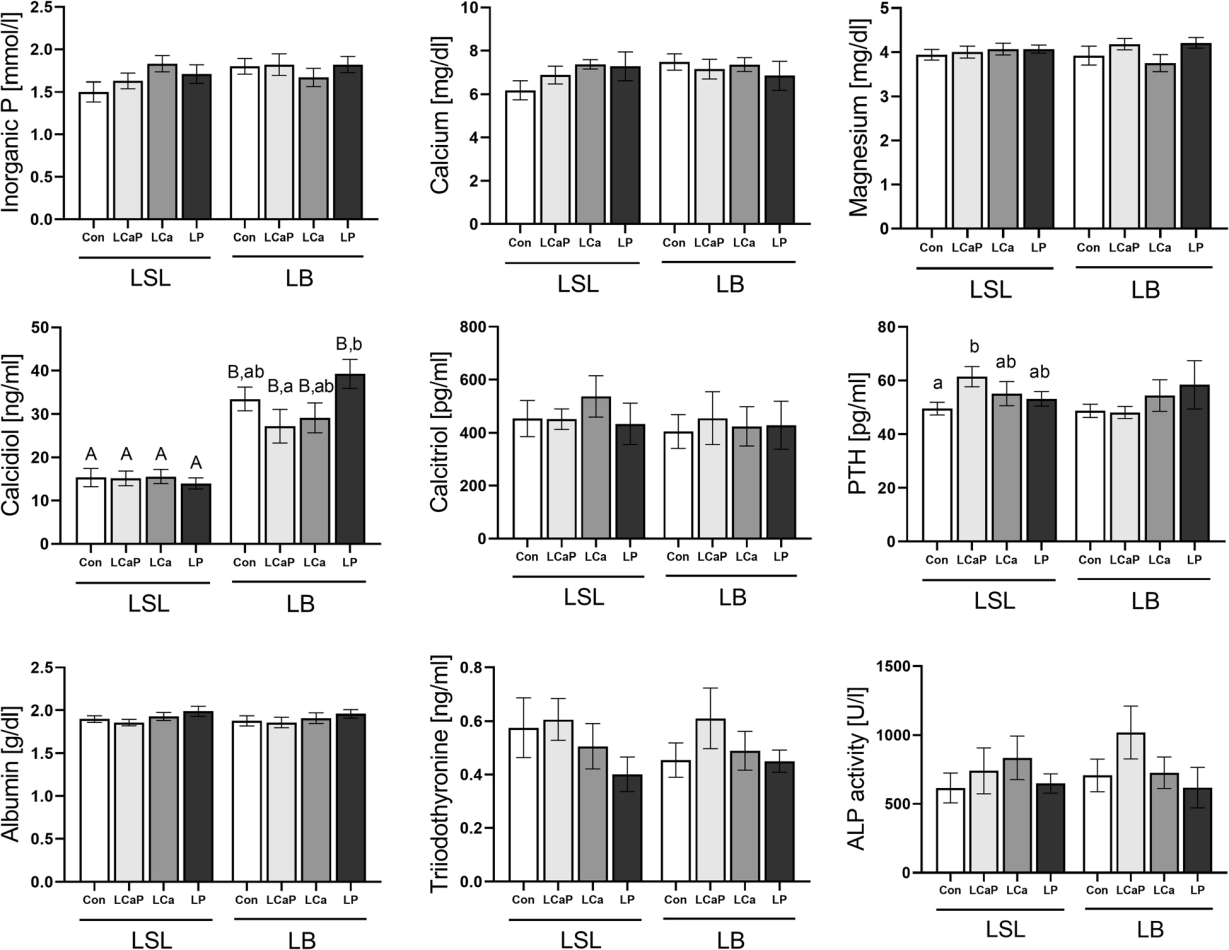
Plasma parameters referring to mineral homeostasis and growth of LSL and LB laying hens fed a standard control diet (Con), reduced Ca and P levels (LCaP), reduced Ca levels (LCa) or reduced P levels (LP). Values are displayed as mean ± SE. Data for inorganic P and Ca were taken from [17]. Superscripts indicate statistical significance (*p* < 0.05) between hen strains as capital letters or within strains between dietary groups as small letters

Average body weight differed significantly between strains but not between diet groups within strain. The dietary groups comprised the control group (Con) and groups with diets low in Ca and P (LCaP), low in Ca (LCa), and low in P (LP). The average body weights (LSmeans) at slaughter (week 31) for LSL were 1648 g (Con), 1683 g (LCaP), 1641 g (LCa), and 1599 g (LP) [19]. The corresponding body weights (LSmeans) of LB animals were 1784 g (Con), 1809 g (LCaP), 1929 g (LCa), and 1838 g (LP). Statistical analysis of zootechnical data of these birds was performed by Sommerfeld et al. 2020 [19] and revealed that body weight was significantly higher in LCa hens of the LB strain compared to Con animals. Plasma levels of albumin, magnesium, T3 and activity of alkaline phosphatase were not significantly different between treatments (*p* > 0.05, Fig. 1). For vitamin D, levels of the storage form calcidiol were significantly higher in LB compared to LSL across all dietary groups (*p* ≤ 0.03). Moreover, significant differences in calcidiol concentrations were observed for LCaP (39.26 ± 3.35 ng/ml) compared to LP (39.26 ± 3.35 ng/ml) in LB (*p* = 0.03). Plasma concentrations of the active form, 1,25(OH) vitamin D (calcitriol), showed a high individual variability and did not differ significantly between treatments (*p* > 0.05). While PTH levels were not affected by diet in LB hens, it was significantly higher under the LCaP diet than the Con diet in LSL hens (*p* = 0.04).

**Fig. 2:**
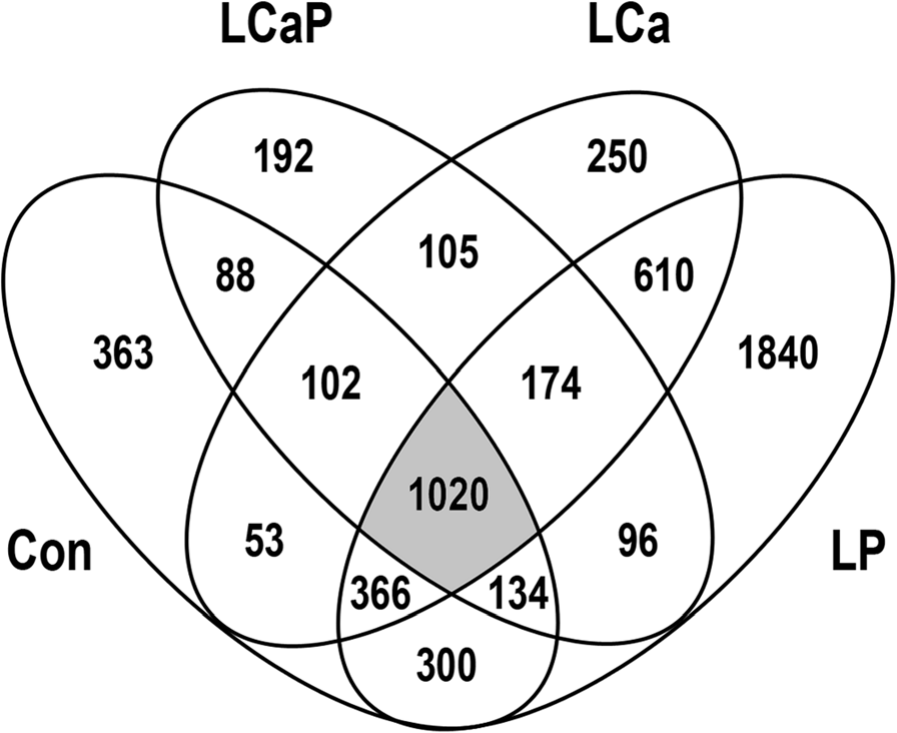
Venn diagram of differentially expressed genes identified in diet-specific comparisons between LSL and LB laying hens fed a standard control diet (Con), reduced Ca and P content (LCaP), reduced Ca content (LCa) or reduced P content (LP). The 1020 DEGs that are present in all dietary comparisons represent the laying hen strain-specific transcriptional patterns in the jejunum

**Fig. 3:**
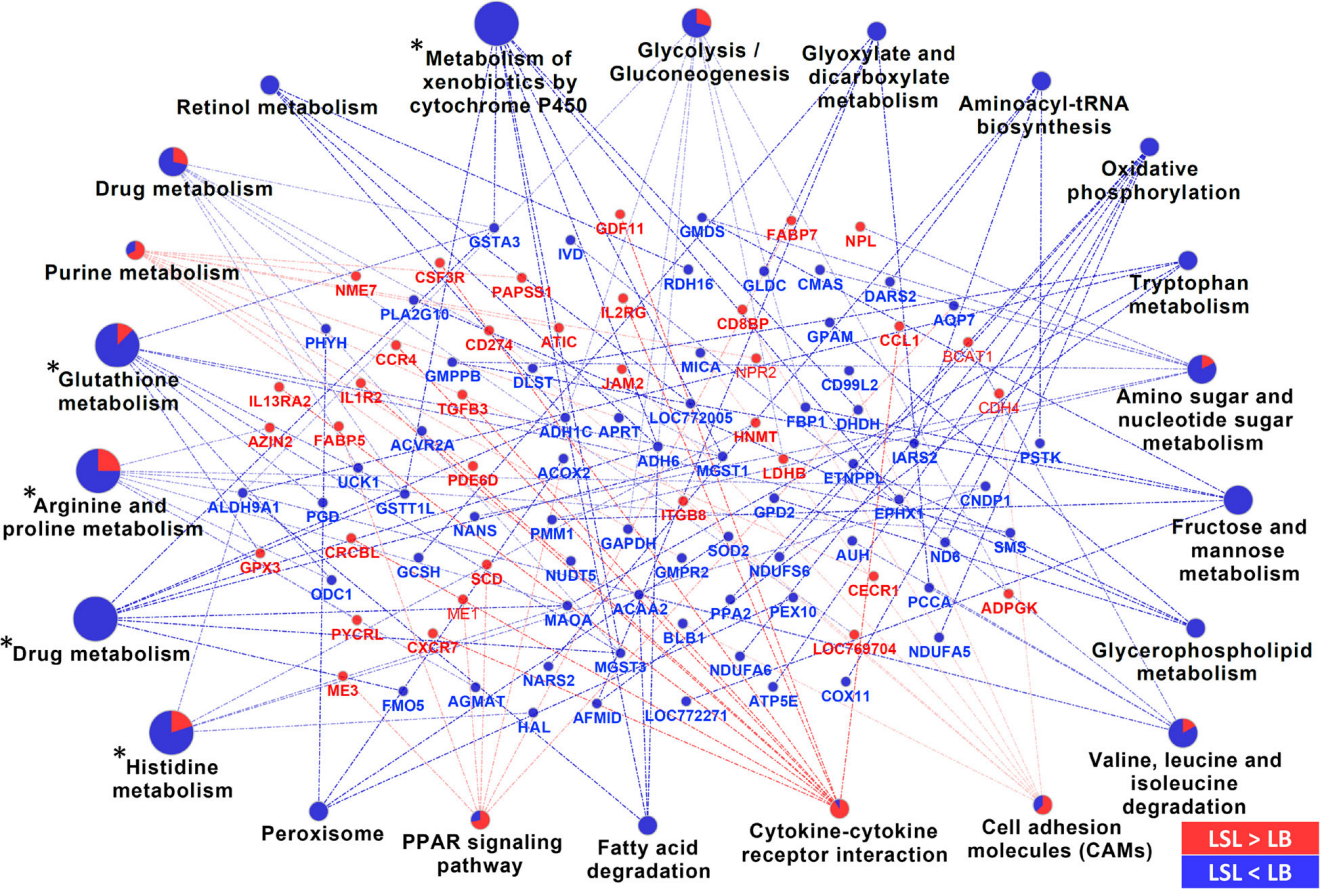
KEGG pathway analysis of 1020 genes found to be consistently differentially expressed between LSL and LB strains covering all four dietary comparisons. The size of the pathway term represents the term p-value. Terms indicated by an asterisk were considered significant (Benjamini-Hochberg adjusted p-value < 0.05)

The comparison of expression profiles of LSL and LB in the four experimental diets by the base model showed considerable differences between laying hen strains (Fig. 2, Additional file 1). For the Con diet, the comparison between strains revealed 2426 differentially expressed genes (DEGs; *p* < 0.01, *p*_adj_ < 0.029). For the LCaP diet, 1911 DEGs (*p* < 0.01, *p*_adj_ < 0.041) were identified between LSL and LB. The diets reduced in either Ca or P resulted in a number of 2680 (*p* < 0.01, *p*_adj_ < 0.028) and 4540 DEGs (*p* < 0.01, *p*_adj_ < 0.012) between strains, respectively. The intersection of the strain comparisons for all four dietary groups revealed 1020 genes, which are considered to represent the strain-specific differences in jejunal nutrient utilization and metabolism. Interestingly, all 1020 DEGs showed a consistent expression pattern in terms of their mRNA abundances over all four diets, with 527 upregulated (LSL > LB) and 493 downregulated (LSL < LB) genes. This information was subjected to pathway enrichment analysis using the KEGG database, highlighting those genes out of the 1020 that accumulate in certain pathways (Fig. 3). Enriched pathways considering the significance threshold (*p*_adj_ < 0.05) include ‘metabolism of xenobiotics by cytochrome P450’, ‘glutathione metabolism’, ‘arginine and proline metabolism’, ‘drug metabolism’, and ‘histidine metabolism‘ (Fig. 3).

**Fig. 4:**
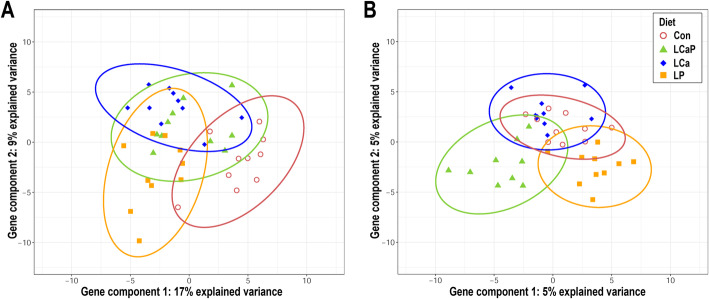
Principal component analysis of the LSL (**A**) and LB (**B**) laying hens fed a standard control diet (Con), reduced Ca and P levels (LCaP), reduced Ca levels (LCa) or reduced P levels (LP). The plots represent the first two components, which are derived from a variable selection approach (sPLS-DA) based on jejunal gene expression profiles

After filtering, 13,123 and 12,703 genes were included in the analysis for contrasting diets within LSL and LB, respectively. The variable selection approach based on gene expression data revealed no clear separation of all four groups within a hen strain (Fig. 4). However, in LSL the LP group is partly separated from the Con group in the first component, which explained 17 % of the variance. Samples of the LCa and LCaP groups largely overlapped. For LB, the LP group was found to be separated to some extent from the LCaP group when the first component was considered. Moreover, these two dietary groups were partly distinct from Con and LCa, which largely overlap on the two components. Correspondingly, the differential gene expression analysis revealed a considerable number of DEGs exclusively for the Con – LP contrast in LSL and the LCaP – LP contrast in LB (Additional file 1). For Con – LP, 503 DEGs were detected in LSL. For LB, the contrast between LCaP and LP revealed the highest number of DEG with in total 568 genes. The comparison of expression profiles of other groups within each strain revealed in only minor alterations at the transcriptional level (Additional file 1).

**Fig. 5:**
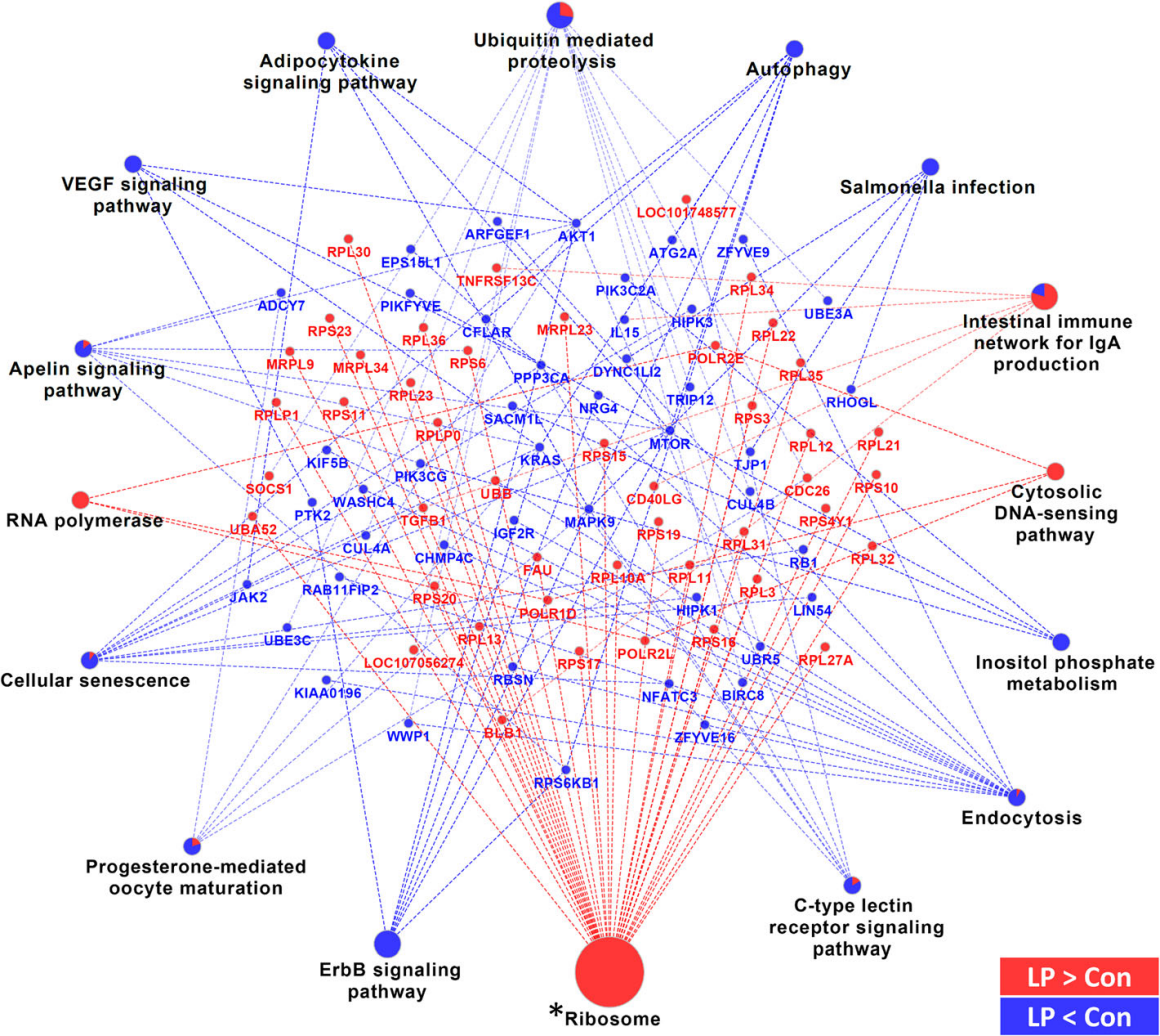
KEGG pathway analysis of genes found to be differentially expressed between Con and LP hens of the LSL strain. The size of the pathway designation represents the p-value. Terms indicated by an asterisk were considered significant (Benjamini-Hochberg adjusted p-value < 0.05)

**Fig. 6:**
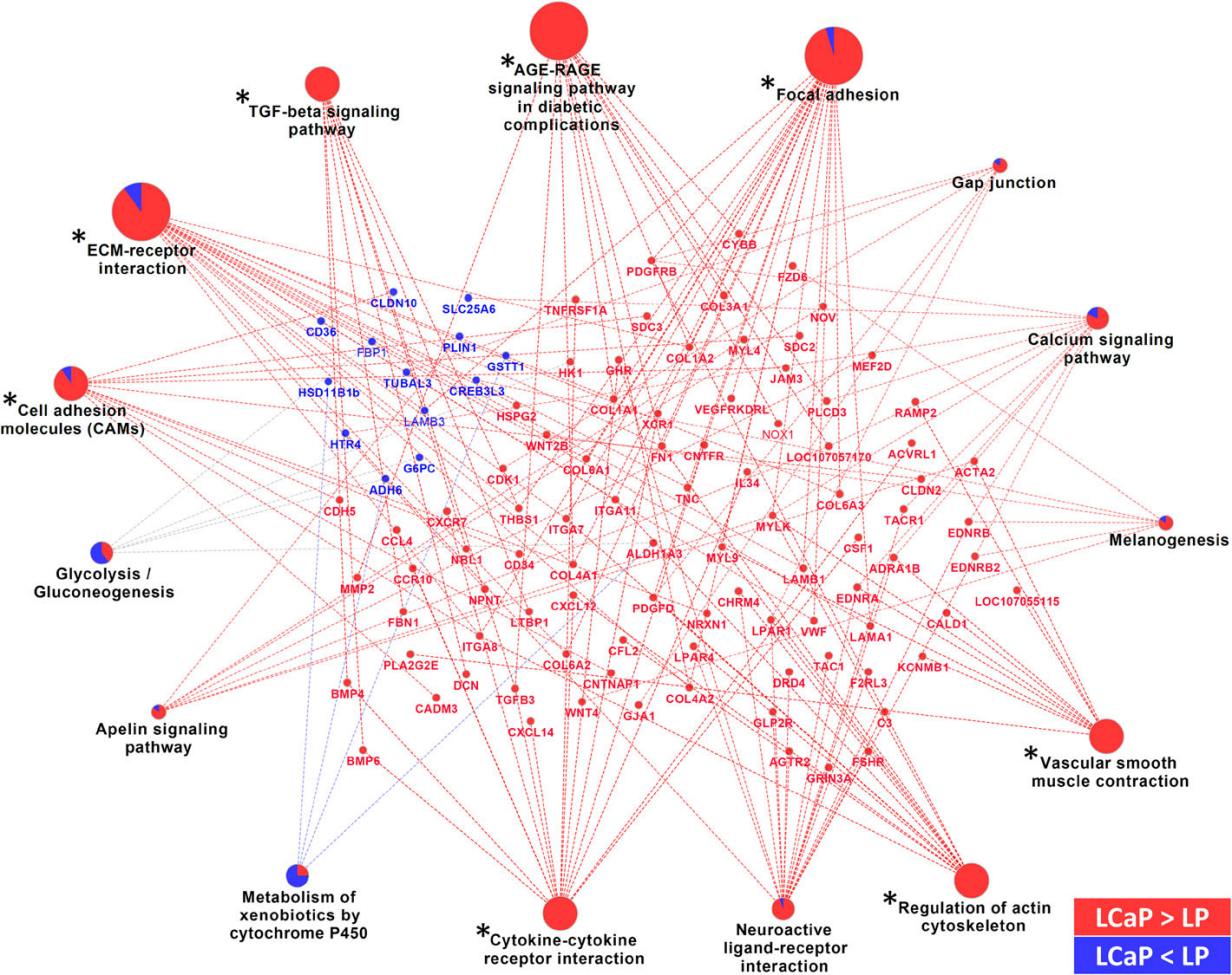
KEGG pathway analysis of genes found to be differentially expressed between LCaP and LP hens of the LB strain. The size of the pathway designation represents the p-value. Terms indicated by an asterisk were considered significant (Benjamini-Hochberg adjusted p-value < 0.05)

Based on the DEGs identified in reasonable numbers for the contrasts Con-LP in the LSL strain and LCaP-LP in the LB strain, biological pathway analyses were performed using the KEGG database (Figs. 5 and 6). For the contrast between Con and LP in LSL, the ‘ribosome’ pathway was found to be enriched (*p*_adj_ < 0.05; Fig. 5). Three pathways including ‘intestinal immune network for IgA production’ (*p*_adj_ = 0.05), ‘ubiquitin mediated proteolysis’ (*p*_adj_ = 0.06) and ‘ErbB signaling pathway’ (*p*_adj_ = 0.10) tended to be significantly enriched (Fig. 5). DEGs contributing to the ‘ribosome’ pathways were entirely upregulated in the LP group compared to control animals (LP > Con), whereas for ‘ubiquitin mediated proteolysis’ the majority of genes were lower abundant in LP chicken compared to Con animals. Thematically overlapping pathways were identified using IPA (Table 1). Predicted activation state (z-score) of these pathways revealed a significant activation of ‘EIF2 signaling’ (z-score = -3.0) in the LP chickens compared to Con, whereas for the ‘regulation of eIF4 and p70S6K signaling’ and ‘mTOR signaling’ a trend for inhibition of this pathways was observed in the LP group (z-scores = 1.89). In addition, an enrichment of genes in ‘IL-4 signaling’ and ‘glucocorticoid receptor signaling’ was identified.

**Table 1:** Canonical pathways enriched by DEGs comparing Con and LP groups of LSL laying hens and LCaP and LP of LB laying hens

Canonical pathways	*p*_adj_-value	z-score*	Molecules
*Lohmann Selected Leghorn (LSL): Con vs. LP*			
EIF2 Signaling	< 0.001	3.00	AKT1,ATF3,EIF3K,FAU,KRAS,PAIP1,PIK3C2A,PIK3CG, RPL10A,RPL11,RPL12,RPL13,RPL21,RPL22,RPL23,RPL27A, RPL3,RPL30,RPL31,RPL32,RPL34,RPL35,RPL36,RPLP0, RPLP1,RPS10,RPS11,RPS15,RPS16,RPS17,RPS19,RPS20, RPS23,RPS3,RPS4X,RPS6,UBA52,XIAP
Regulation of eIF4 and p70S6K Signaling	< 0.001	-1.89	AKT1,EIF3K,EIF4EBP2,FAU,KRAS,MTOR,PAIP1,PIK3C2A, PIK3CG,RPS10,RPS11,RPS15,RPS16,RPS17,RPS19, RPS20,RPS23,RPS3,RPS4X,RPS6,RPS6KB1
mTOR Signaling	0.005	-1.89	AKT1,EIF3K,FAU,KRAS,MTOR,PIK3C2A,PIK3CG,PRR5L, RPS10,RPS11,RPS15,RPS16,RPS17,RPS19,RPS20,RPS23, RPS3,RPS4X,RPS6,RPS6KB1
IL-4 Signaling	0.013		AKT1,JAK2,KRAS,MTOR,NFAT5,NFATC3,NR3C2, PIK3C2A,PIK3CG,RPS6KB1,SOCS1
Glucocorticoid Receptor Signaling	0.034		AKT1,BAG1,GTF2A1,GTF2H1,JAK2,KAT2B,KRAS,MAPK9, MED14,NCOA2,NFAT5,NFATC3,NR3C2,PIK3C2A,PIK3CG, POLR2E,POLR2L,PPP3CA,TAF4,TGFB1,YWHAH
*Lohmann Brown (LB): LCaP vs. LP*			
Agranulocyte Adhesion and Diapedesis	< 0.001		ACTA2,CCL23,CD34,CDH5,CLDN10,CLDN2,CXCL12, CXCL14,FN1,JAM3,MMP10,MMP11,MMP17,MMP2, MMP9,MYH11,MYL4,MYL9,PODXL,PPBP,TNFRSF1A
Granulocyte Adhesion and Diapedesis	< 0.001		CCL23,CDH5,CLDN10,CLDN2,CXCL12,CXCL14,JAM3, MMP10,MMP11,MMP17,MMP2,MMP9,PPBP,SDC2, SDC3,THY1,TNFRSF1A
GP6 Signaling Pathway	< 0.001	3.77	COL13A1,COL16A1,COL18A1,COL1A1,COL1A2,COL3A1, COL4A1,COL4A2,COL4A6,COL5A1,COL5A2,COL6A1, COL6A2,COL6A3,LAMA1,LAMB1,LAMB3,NOX1
Axonal Guidance Signaling	< 0.001		ADAM19,ADAMTS1,ADAMTS13,ADAMTS9,BMP4,BMP6, CFL2,CXCL12,ECEL1,EFNB1,EPHB2,FZD6,GNB1L,HHIP, LINGO1,MMP10,MMP11,MMP17,MMP2,MMP9,MYL4, MYL9,NRP2,NTN3,NTN4,PDGFD,PLCD3,PLXND1,PTCH2, RASD2,SDC2,SEMA3F,TUBA4A,UNC5B,UNC5C,WNT2B, WNT4
Inhibition of Matrix Metalloproteases	< 0.001	-1.13	HSPG2,MMP10,MMP11,MMP17,MMP2,MMP9,SDC2, TFPI2,TIMP2
Apelin Liver Signaling Pathway	0.002	2.45	COL18A1,COL1A1,COL1A2,COL3A1,IRS1,PDGFRB
Intrinsic Prothrombin Activation Pathway	0.014	1.34	COL18A1,COL1A1,COL1A2,COL3A1,PROS1
Inhibition of Angiogenesis by TSP1	0.029	0.00	CD36,HSPG2,MMP9,NOS3,SDC2,THBS1
Leukocyte Extravasation Signaling	0.044	1.90	ACTA2,CDH5,CLDN10,CLDN2,CXCL12,CYBB,JAM3,MMP10, MMP11,MMP17,MMP2,MMP9,NOX1,THY1,TIMP2

* z-score: pathways with an absolute z-score ≥ 2 were considered significant. Positive and negative values indicate activation (in LSL: LP > Con; in LB: LCaP > LP) and inhibition (in LSL: LP < Con; in LB: LCaP < P)

For the comparison of LCaP and LP groups in LB chickens, ‘focal adhesion’ was found to be the most enriched pathway with 22 involved genes (Fig. 6). The majority of DEGs in this pathway were found to be more abundant in the LCaP than in LP group. In general, most of the DEGs were more abundant in the LCaP chickens compared to LP animals. Other enriched pathways and corresponding DEGs including ‘cell adhesion molecules’, ‘extracellular matrix interactions’, and ‘regulation of actin cytoskeleton’ are summarized in Fig. 6. For IPA, the DEGs were found to be significantly enriched in nine canonical pathways (Table 1). Among these, the ‘GP6 signaling pathway’ and the ‘Apelin Liver Signaling Pathway’ were predicted to be activated in the LCaP group compared to the LP group. Overall, the results point to an involvement of DEGs in pathways of inflammation, cell adhesion, and extracellular matrix formation.

## Discussion

For many decades, the Ca and P requirements of laying hens have been an important research subject to ensure laying performance [20, 21], it receives additional attention due to the intention to preserve mineral resources in animal-based food production [22]. The complex dynamics of Ca and P metabolism impede the precise assessment of the dietary Ca and P supply in respect to genetics and age [23, 24]. Current dietary recommendations for laying hens range between 32 and 44 g/kg for Ca and between 1.5 and 4.5 g/kg for non-phytate P, depending on age [25]. Interestingly, the requirements for Ca and P of LSL and LB strains are currently assumed identical, although there are significant differences between the strains regarding traits related to mineral utilization. The LB hens have been reported to exhibit a higher bone mass and a higher breaking strength of humeral and tibia bones compared to LSL, whereas bone density was unaffected [17, 26]. However, Khanal et al. observed that with high Ca content in the pre-lay diet, LSL had higher femur mineral density, ash content and breaking strength at the onset of the laying period compared to LB [27]. They concluded that LSL hens have a higher capacity than LB hens to accumulate excess feed Ca in the bones. Obviously, the utilization of micronutrients in LSL and LB hens is based on strain-specific sophisticated metabolic routes, a fact which has been demonstrated for e.g. lysine [28]. Results of the current layer hen trial suggest similar conclusions for Ca and P. Although the analyses demonstrated unaffected plasma Ca and P levels between the strains [19], a clear and marked strain-specific effect was observed for plasma calcidiol (LB > LSL). The difference in the vitamin D system is found exclusively at the level of the respective storage form and is not reflected at the level of the active calcitriol. Due to the required hydroxylation process for the synthesis of calcidiol, this indicates the liver as an important target tissue to initiate local and systemic responses to maintain mineral homeostasis.

The study implies different mineral requirements in LSL and LB, which is substantiated by the fact that differences in gene expression patterns of jejunum were identified in different contrasts between the two strains. While low P supply in LSL induced considerable transcriptional responses compared to Con, hens of the LB strain showed marked differences under low P supply depending on the Ca supply. LSL were reported to have an increased demand and utilization of Ca resulting in eggs with higher eggshell weights compared to LB [29]. Moreover, the regulation of several genes and their products associated with the ‘Ca-ion binding and transport’ and ‘Ca release-activated Ca channels’ establish this pattern in LSL. These adaptive actions of the Ca metabolism might enable LSL to cope better with moderate dietary Ca restrictions than LB hens. However, it needs to be considered that nutritional recommendations for the two strains are identical, while the optimum might be better matched for one than for the other.

For the two strains studied, the dietary reduction of Ca and/or P was reflected to varying degrees in the gene expression patterns in the gut. For the LSL strain, marginal differences were observed for the group comparisons Con vs. LCaP and Con vs. LCa, whereas the reduction of dietary P supply resulted in considerable changes in intestinal gene expression compared to the control. These findings suggest that the animals investigated were generally able to adapt to the dietary changes by endogenous mechanisms. This was also reflected in plasma levels and performance traits, which were found to be mostly inconspicuous compared to Con, although body weight was significantly increased in hens of the LB strain submitted to a low Ca [19]. Merely increased plasma PTH levels in the LCaP group compared to Con point to active adaptation mechanisms to maintain Ca and P homeostasis. Indeed, PTH is an important regulator of osteoclast activity and may mediate intensified bone resorption in the LCaP group of LSL hens in order to mobilize additional Ca and P [30].

For LSL, the dominant dietary treatment in terms of identified intestinal DEGs was the lowered P diet (LP group) in comparison to Con. The integration of functional annotations of corresponding DEGs showed that the main pathways initiated by LP treatment include ribosomal protein synthesis and the regulation of cellular signaling cascades. In accordance, the protein biosynthesis pathway was recently found to be affected in the intestinal epithelium of Japanese quail with divergent P utilization efficiency [31]. In this quail study, high P utilization efficiency was ascribed to an accelerated cell proliferation in the intestine. The current list of DEGs further revealed an impairment of the mTOR signaling pathway, which has important functions in cell proliferation, differentiation, growth, and metabolism [32]. These differences are mainly driven by the differential abundance of ribosomal proteins and ubiquitin proteins. Effects of the diet composition on proliferation of intestinal cells are described for several nutritional components including non-starch polysaccharides, short chain fatty acids, and vitamins [33]. Non-starch polysaccharides affect viscosity of digesta in the intestine and induce renewal of the epithelium due to delayed nutrient absorption. Moreover, there are also effects of dietary mineral supplements including Ca and P described to affect intestinal cell proliferation in rodents [34].

Among the differentially abundant genes, a number of transcripts encoding for transport proteins were obtained. These comprised anion transmembrane transporters (*SLC4A4*, *SLC4A8*, and *SLC35B3*) as well as some molecules involved in mineral homeostasis such as *ATP2C1* and *CASK*. ATP2C1 regulates Ca concentrations in cytosol and plays an important role for protein synthesis in the endoplasmic reticulum [35]. *CASK* encodes for a Ca/calmodulin dependent serine protein kinase mediating intracellular effects downstream of plasma membrane Ca pumps [36]. No effects on the most common transcellular Ca and P transporters were identified between LP and Con animals of LSL. However, paracellular transport processes might be affected through changes in *TJP1* and *ADAM10* expression. Both genes affect cell-to-cell adhesion and influence the mobility of molecules in the paracellular area [37, 38]. Consequently, the lower abundance of *TJP1* in LP compared to Con might increase the selective permeability for ions but conversely might also increase the risk of transferring intestinal microbes, toxins, and antigens into tissues [39]. In fact, intestinal signaling pathways related to the immune response, in particular ‘intestinal immune network for IgA production’ (KEGG) and ‘IL-4 signaling’ (IPA), were considered influenced in the comparison of Con and LP through the functional enrichment analysis. IgA production relies on antibodies that are produced on the basis of bacterial antigens and that aim to counteract toxins and pathogenic microbes at the contact surface between host and intestinal microbiota [40]. In chickens identified to be more resistant to *Salmonella* infections, it was found that the pathway of IgA production was activated compared to more susceptible individuals [41]. However, any change in the dietary composition, even changes in individual minerals, drives alterations of the intestinal microbial community and requires an adaptation of the host’s defence mechanisms [42, 43].

In LB hens, the plasma calcidiol levels were higher compared to LSL hens. Moreover, higher calcidiol levels were found in LB hens fed LP compared to LCaP diets. Plasma levels of calcidiol reflect the vitamin D status, which is affected by long-term feed supply and individual vitamin D metabolism (e.g. hepatic hydroxylation processes, renal clearance), since under current housing conditions UV-mediated endogenous vitamin D synthesis is not present in laying hens [44]. Thus, LB hens fed the LCaP diet might use a higher proportion of the available vitamin D to counteract mineral shortage and maintain mineral homeostasis. The analyses of dietary treatments in the LB strain revealed increased expression of genes involved in ‘GP6 signaling’ and ‘Focal adhesion’ in LCaP compared to LP fed laying hens. A considerable number of DEGs encoding collagens and matrix metalloproteinases (MMP) have been identified. Indeed, the GP6 proteins are major signaling receptors in collagen formation and function, which together with integrins, tenascins, fibronectins and laminins constitute the main components of the extracellular matrix (ECM) [45, 46]. The MMP are a family of zinc-dependent proteinases that are secreted to the extracellular space or localized to the cell surface in a premature state. Once activated, MMP are collectively able to cleave all components of the ECM [47]. Focal adhesions constitute a large macromolecular assembly of proteins namely vinculin, talin, paxillin, zyxin, and α-actinin that associate with integrins in order to facilitate the anchorage of the cell and the ECM but also to support cell migration [48, 49]. The recruited cellular components that form a focal adhesion remain anchored to the ECM to allow sequential cell migration, which is of particular importance for intestinal enterocytes and immune cells [50]. Indeed, the DEGs assigned to both agranulocyte and granulocyte adhesion pathways are responsible for facilitating the migration of immunocompetent cells in the intestinal endothelium. Taken together, the results obtained for the comparison of LCaP with LP diets in LB hens point to a number of DEGs encoding collagens and MMPs that might have an impact on ECM formation. Apparently, the amounts of dietary Ca and P have to be considered to trigger structural changes in the intestinal epithelium. In fact, a previous jejunal transcriptome study in broilers linked the increased expression of genes encoding for collagen and ECM to increased villus length for improved nutrient uptake and energy utilization [51]. Results in the present study suggest therefore a vulnerability of LB hens for inadequate Ca intake. Further analysis of the microanatomy in laying hens are required to map potential phenotypic adaptations related to the maintenance of mineral homeostasis. A limitation of the study is that although the sampling times of the current study were standardised and largely controlled, the calcium requirement of egg-laying hens, which is hormonally regulated, varies considerably depending on oviposition and the circadian cycle [52]. To some extent, this could also influence gene expression patterns in the gastrointestinal tract, as the demands on the entire organism shift due to the higher calcium requirement for eggshell calcification processes [53].

## Conclusions

Differences between LSL and LB hens, which are present in genetic and morphological aspects, are also reflected in the transcriptional profile of the jejunum. Accordingly, the response to varying levels of dietary Ca and P concentrations differs in the two laying hen strains. LSL hens might be more prone to effects of varying dietary mineral supply on intestinal cell proliferation rate, while adaptive responses occur at the level of the paracellular transport and immune competence. The endogenous mechanisms in LB hens might involve the formation of extracellular matrix for compensatory improvement of the absorptive capacity. The results of the current study indicate that LSL and LB laying hens have different mineral and vitamin D requirements owing to different transcriptome, which potentially might be exploited to reduce mineral resources. In terms of environmental protection and poultry management, there is both a need and a possibility to further specify the requirements for dietary Ca, P, and vitamin D supply in LSL and LB laying hen strains.

## Methods

### Birds and diets

The animal trial was conducted at the Agricultural Experiment Station of the University of Hohenheim, Germany, and was approved by the Regierungspräsidium Tübingen, Germany (HOH50/17TE). Procedures were in accordance with the German Animal Welfare Legislation.

The trial is based on two modern strains of laying hens supplied with variable Ca and P levels [19]. In particular, for each of the strains Lohmann Selected Leghorn (LSL, *n* = 40) and Lohmann Brown (LB, *n* = 40; Lohmann Tierzucht GmbH, Cuxhaven, Germany), four dietary groups (*n* = 10 per group) with varying Ca levels and non-phytate P levels (standard vs. reduced) were formulated as described previously [19]. In brief, except for Ca and P levels the composition of the corn-soybean based diets met current recommendations [54]. Differences in Ca and P levels were achieved by varying levels of mineral monocalcium phosphate and limestone. The analyzed Ca and total P levels of the control diet (Con) were 39.5 g/kg dry matter (DM) and 5.3 g/kg DM. The treatment groups were supplied with diets low in Ca and P (LCaP), low in Ca (LCa), and low in P (LP). The retrieved dietary Ca and P contents were 34.4 g/kg DM and 4.7 g/kg DM for LCaP, 35.1 g/kg DM and 5.3 g/kg DM for LCa, and 40.3 g/kg DM and 4.7 g/kg DM for LP diets, respectively. The feed was formulated to minimize plant based phytases and no additional phytases of microbial origin were added. For each of the two strains LSL and LB, the four diet groups consisted of 10 birds each, with the progeny of the same 10 fathers in each of the groups. Birds were group-housed in pens for the first 26 weeks and then were rehoused individually into metabolism unity cages in a randomized complete block design, which resulted in 10 replicates per strain and diet. The feeding trial lasted 21 days in metabolism units in which each bird was fed individually. Chickens were phenotyped for zootechnical and physiological parameters as described elsewhere [19]. At the age of 31 weeks, the hens were stunned and subsequently slaughtered by exsanguination Prior to slaughter, each bird was subjected to a two-hour feed withdrawal period, followed by a one-hour re-feeding period. Sampling started at 9 am, with twenty birds slaughtered at 15-minute intervals on each of four consecutive days, with the order of slaughter recorded. Trunk blood was collected in heparin-containing tubes and centrifuged to obtain plasma samples (10 min at 2500 × g). Samples were stored at -80 °C until analysis of plasma parameters. Approximately 3 cm distal to the duodenal loop, a jejunum sample of 2 cm in length was collected from each bird.The mucosa was rinsed with 0.9 % NaCl solution and then scraped off with a scalpel. Samples were frozen on dry ice and stored at -80 °C upon RNA extraction.

### Measurement of blood parameters

The plasma samples collected at slaughter were analysed to measure the levels of albumin, magnesium and alkaline phosphatase activity using commercial assays via the Fuji DriChem 4000i according to manufacturer’s instructions (FujiFilm, Minato, Japan). Hormone measurements were performed in duplicate with commercially available enzyme-linked immunosorbent assays (ELISA). Corresponing kits were processed according to manufacturer’s instructions for parathyroid hormone (CSB-E118880Ch, CusaBio, Houston, USA), triiodothyronine (EIA-4569, DRG, Marburg, Germany), 25(OH) vitamin D (EIA-5396, DRG), and 1,25(OH) vitamin D (AC-62F1, Immunodiagnostic Systems GmbH, Frankfurt am Main, Germany). For statistical analysis of the mentioned blood parameters and hormones, a linear model was applied including dietary group, laying hen strain, hen father and slaughter order (R language, version 3.6.2, package stats). Differences were considered significant at *P* ≤ 0.05.

### RNA extraction and sequencing

Total RNA was extracted from all 80 jejunal samples using TRIzol Reagent (Invitrogen, Karlsruhe, Germany) according to the manufacturer’s protocol. Subsequently, mRNA was extracted using the Rneasy Mini Spin kit including an additional Dnase digestion (Qiagen). The quantity and quality of final mRNA was determinded using NanoDrop ND-2000 (Peqlab, Erlangen, Germany) and Bioanalyzer 2100 devices (Agilent Technologies, Waldbronn, Germany). RNA integrity numbers were between 7.1 and 9.4. Sequencing libraries were prepared with the stranded mRNA library preparation kit (Illumina, San Diego, CA, USA). Sample-specific, tagged libraries (*n* = 80) were pooled and sequenced on the Illumina HiSeq 2500 in a paired-end setup with 2 × 101 bp reads. The corresponding raw data were deposited in the EMBL-EBI (https://www.ebi.ac.uk) database under accession number E-MTAB-9109.

### Sequencing data processing and gene expression analysis

Initially, raw sequencing reads were checked for quality and preprocessed using FastQC (version 0.11.7) and Trim Galore (version 0.5.0; https://www.bioinformatics.babraham.ac.uk/projects/). Reads with low quality (mean Q-score < 20) and short length (< 30 bp) were filtered out. Remaining reads were mapped to the current chicken genome assembley (GRCg6a, Ensembl release 95) using Hisat2 (version 2.1.0; http://daehwankimlab.github.io/hisat2/). Gene-specific read counts were extracted with HTseq (version 0.11.2; https://htseq.readthedocs.io/en/master/). Differentially expressed genes (DEG) between the experimental groups were obtained using DESeq2 (DOI: 10.18129/B9.bioc.DESeq2). Initially, outlier detection approaches, including the distance between individual data sets, the distribution of signal intensities and the quality and quantity of the individual data were assessed using the arrayQualityMetrics R package (DOI: 10.18129/B9.bioc.arrayQualityMetrics). One sample (from LB LCaP group) was excluded from analysis due to low sequencing depth (Additional file 2). The count data were filtered to retain only genes with observations of 5 or more counts in at least 10 animals. Firstly, a base model to identify DEGs between the two laying hen strains within each dietary group was performed. Secondly, a statistical model was designed to reveal DEGs in the contrasts of diets within each of the two strains. This model included hen father as fixed effect to account for genetic relationship of animals. Genes were considered as significantly differentially expressed meeting the criteria of *p*-value < 0.01 and Benjamini-Hochberg adjusted *p*-value < 0.15. Normalized count data was further used to select the most important genes to distinguish between dietary groups using the sparse Partial Least Squares discriminant analysis (sPLS-DA) function of the mixOmics R package [55]. The variable selection approach considered the first two components with 50 variables each. The differentiation of groups was presented in a scatter plot. KEGG pathway enrichment analysis of the identified DEGs was performed using Cytoscape software (version 3.6.1) with the ClueGO plugin (version 2.5.1). The Clue GO plug-in generates functionally clustered KEGG annotation Networks for a list of DEGs. The *p*-values were calculated by right-sided hypergeometric tests and Benjamini-Hochberg adjustment was used for multiple testing correction. KEGG pathways with an adjusted *p*-value < 0.05 and comprising at least five DEGs were considered significant.

## Supplementary information

Additional file 1 Differentially expressed genes in the jejunum mucosa in LSL and LB laying hens following the variations of dietary calcium and phosphorus levels. Associated statistics refer to strain or dietary effects (base mean – average of the normalized counts over all samples included in the respective analysis, *p*-value, Benjamini-Hochberg adjusted *p*-value, fold change).

Additional file 2 Sequencing statistics, including read count and number of mapped reads for the jejunum samples used for mRNA sequencing.

## Data Availability

shttps://www.ebi.ac.uk) under accession number E-MTAB-9109. Information on the chicken genome assembly (GRCg6a, Ensembl release 95), which was considered for read mapping and gene annotation (e.g., gene identifiers in Additional file 1), is accessible in the Ensembl database (http://ftp.ensembl.org/pub/release-95/fasta/gallus_gallus/).

## Abbreviations

Ca: Calcium
CASR: Ca-sensing receptor
Con: experimental group `Control´
DEG: Differentially expressed genes
DM: dry matter
ELISA: enzyme-linked immunosorbent assays
FDR: false discovery rate
FGF23: fibroblast growth factor 23
GO: gene ontology
KEGG: Kyoto Encyclopedia of Genes and Genomes
LB: Lohmann Brown
LCa: experimental group `low Ca´
LCaP: experimental group `low Ca and P´
LP: experimental group `low P´
LSL: Lohmann Selected Leghorn
P: Phosphorus
PTH: parathyroid hormone
sPLS-DA: sparse Partial Least Squares discriminant analysis
VDRE: vitamin D response elements

## Declarations

**Ethics approval and consent to participate**

The animal trial was approved by the local authority, the Regierungspräsidium Tübingen, Germany (HOH50/17TE) and performed at the Agricultural Experiment Station of the University of Hohenheim, Germany. All procedures were in accordance with the German Animal Welfare Legislation and complied with the European Union Directive 2010/63/EU on the protection of animals used for scientific purposes.
